# The significance of sleep quality in euthymic bipolar patients from Nigeria

**DOI:** 10.4102/sajpsychiatry.v28i0.1739

**Published:** 2022-02-16

**Authors:** Oluyomi Esan, Ayodele Fela-Thomas

**Affiliations:** 1 Department of Psychiatry, Faculty of Clinical Sciences, University of Ibadan, Ibadan, Nigeria; 2 Federal Neuro Psychiatric Hospital, Benin City, Nigeria

**Keywords:** sleep quality, euthymic, remission, bipolar disorder, Nigeria

## Abstract

**Background:**

Bipolar disorder is highly under-researched in Africa. Existing studies show that racial/ethnic disparities exist for sleep quality. Poor sleep quality in bipolar disorder causes significant morbidity and mortality even during periods of euthymia.

**Aim:**

This study aimed to assess sleep quality and its correlates amongst euthymic patients with bipolar I disorder from Nigeria.

**Setting:**

The study was carried out in a teaching hospital, and state hospital, in Ibadan, Nigeria.

**Method:**

This cross-sectional study was conducted amongst 76 euthymic bipolar patients aged between 18 and 60 years, meeting the Diagnostic and Statistical Manual of Mental Disorders, Fourth Edition (DSM-IV) diagnostic criteria for bipolar disorder. Euthymia was defined as having a score of ≤ 5 on the Young Mania Rating Scale and < 8 on the Hamilton Depression Rating Scale. Sleep quality was assessed with the Pittsburgh Sleep Quality Index (PSQI).

**Results:**

A total of 37 (48.7%) participants had poor quality sleep. Sleep quality was associated with marital status (*p* = 0.013) and suicide plan (*p* = 0.047). Participants with good sleep quality had higher total sleep duration, lower time to fall asleep (sleep latency), better subjective quality of sleep, were less likely to use sleep medications and had less daytime dysfunction than participants with poor sleep quality. All *p*-values were < 0.05. Subjective quality of sleep, ongoing use of sleep medication, daytime dysfunction were independently associated with poor sleep quality.

**Conclusion:**

Poor sleep quality frequently persists during euthymic periods in patients with bipolar disorder. The correlates identified can be targeted for intervention during treatment.

## Introduction

Bipolar disorder is potentially a lifelong episodic and recurrent illness characterised by prominent and persistent mood swings and with a diverse course that can often result in significant distress and impairment in functioning. Sleep disturbances are central to the onset, relapse, recovery, prognosis and outcome of bipolar disorder.^1^ Sleep disturbances occur in all stages of bipolar disorder and play an essential part in the aetiology and maintenance of the disorder including periods of remission.^2^

Contrary to previously held assumptions, remission periods (euthymia) in bipolar disorder are not asymptomatic periods. Such euthymic periods are characterised by psychopathologies including sleep disturbances and poor sleep quality. Such psychopathologies and disturbances produce a more severe clinical outcome that considerably affects the quality of life of affected individuals.^3,4,5^

Poor sleep quality in bipolar disorder causes substantial medical and psychological morbidities such as reduced global functional ability, relapses, recurrences, neurocognitive impairment and poor prognosis.^6,7,8,9^ Poor sleep quality also increases the risk of cardiovascular morbidity (e.g. heart attack), diabetes mellitus, cerebrovascular accidents (strokes), obesity, neurocognitive impairments, unintentional injuries (such as motor vehicle accidents, falls, poisoning, fire/burns, and increased workplace accidents).^10,11,12,13^ Poor sleep quality negatively affects daytime functioning, social functioning, occupational functioning, and other important areas of functioning.^14,15^

Very few studies have been conducted on sleep quality during the euthymic phase of bipolar disorders. Also, very little is known about the correlates and predictors of sleep quality during this period.^16^ The majority of such existing studies have been carried out in non-Caucasian populations. This is concerning since racial/ethnic disparities exist for sleep quality. Furthermore, there is evidence to suggest that sleep quality amongst racial/ethnic minorities is disproportionately understudied.^17^

Understanding the factors associated with sleep quality during the euthymic phase of bipolar I disorder may play a significant role in improving clinical course and help to negotiate a more favourable outcome for patients with bipolar disorder.

This study aimed to assess sleep quality and its correlates amongst well-characterised euthymic patients with bipolar I disorder.

## Subjects and methods

The Mulberry study is a cross-sectional study comparing sociodemographic and clinical characteristics such as neurocognitive function, psychopathology, sleep, physical activity and spirituality between patients with bipolar disorder, patients with schizophrenia and healthy controls.^18^ A complete account of the methodology has been described elsewhere.^18^ The current analysis is restricted to participants with bipolar disorder in the Mulberry study. The study was carried out in a teaching hospital, and state hospital, in Ibadan, Nigeria. Assessments were carried out between February and October 2018 on 76 consecutive and consenting participants with bipolar disorder, aged between 18 and 60 years who were in remission.

### Participants

While waiting to see their doctors, the research assistants personally invited successive patients attending the outpatient psychiatry clinics of the selected hospitals to participate in the study. The study was explained to them and consent was obtained. The study instruments were administered or a date was scheduled during the succeeding week when a detailed interview could be conducted.

### Eligibility

To be eligible for inclusion in the current study, participants had to be euthymic and meet the Diagnostic and Statistical Manual of Mental Disorders, Fourth Edition (DSM-IV) diagnostic criteria for bipolar disorder using the Structured Clinical Interview for DSM-IV Axis I Disorders (SCID-I). A participant with bipolar disorder was deemed to be euthymic if he or she had a score of ≤ 5 on the Young Mania Rating Scale (YMRS) and had a score of < 8 on the Hamilton Depression Rating Scale (HDRS).^19^ All instruments were applied by the trained research assistants.

### Measures

#### Pittsburgh Sleep Quality Index

The Pittsburgh Sleep Quality Index (PSQI) is a self-rated questionnaire designed for use in both research and clinical practice. It assesses the quality of sleep and sleep disturbances over a 1-month time interval.^20^ It has 19 questions, which generates seven component scores. The seven components (1 to 7) are: (1) subjective sleep quality, (2) sleep latency, (3) sleep duration, (4) habitual sleep efficiency, (5) sleep disturbances, (6) use of sleeping medication, and (7) daytime dysfunction. Each component score is rated from 0 to 3. Higher scores mean poorer quality of sleep. The seven component scores are summed up to give a global score that ranges from 0 to 21; the higher the score, the worse the quality of sleep. A global score of > 5 was used as the cut-off score between poor sleep quality and good sleep quality.^14,20^

### Sociodemographic and clinical assessments

Important sociodemographic and clinical information was obtained from all participants. The participants were also assessed with the following instruments:

1. The Young Mania Rating Scale (YMRS)^19^
2. The 17-item Hamilton Depression Rating Scale (HDRS)^21^
3. The Positive and Negative Syndrome Scale (PANSS)^22^

#### Data analysis

We carried out data analysis with the Statistical Package for the Social Science (SPSS), version 22.0 for Windows. Categorical variables were summarised using frequencies and proportions. The chi-squared test was used to analyse the relationship between two qualitative variables. Continuous variables were described using means and standard deviation (s.d.) if they were normal in type or using medians and ranges if not normally distributed. An independent sample *t*-test was used to assess whether there was a difference between two independent sample means, while the Mann-Whitney U test was used to compare differences between two independent groups when the dependent variable was not normally distributed.

### Ethical considerations

The study was conducted following the guidelines laid down in the Declaration of Helsinki. The protocol and procedures were reviewed and approved by the Oyo State Research Ethical Review Committee (AD13/479/746).

## Results

A total of 110 participants with a diagnosis of bipolar I disorder were recruited into the study, however, only 76 met the criteria for euthymia. The 76 participants (47 females, 29 male) were subsequently included in the current analysis. The mean age was 39.09 ± 11.10 years. A total of 37 (48.7%) participants had poor quality sleep during the past 1 month. There was no significant difference in socio-demographic and clinical characteristics between the group with poor sleep quality and the group with good sleep quality except for marital status and lifetime suicidal plan. A significantly higher proportion of the participants who had poor sleep quality were single or never married compared to those with good sleep quality (43.2% vs. 26.3% *p* = 0.013). Also, a significantly higher proportion of participants with poor sleep quality had made a suicide plan compared to those with good sleep quality (24.3% vs. 7.7% *p* = 0.047) (see Tables 1a and 1b).

**TABLE 1a:** The sociodemographic and clinical characteristics of participants and comparison of clinical features of bipolar disease according to Pittsburgh Sleep Quality Index scores.

Characteristics	Mean ± s.d.	Range	Mdn	PSQI ≤ 5 (*n* = 39)	Range	Mdn	PSQI > 5 (*n* = 37)	Range	Mdn	*p*
Age of patient (years)	39.09 ± 11.10	-	-	39.67 ± 11.11	-	-	38.49 ± 11.20	-	-	0.646
Age at onset of first mood disorder episode (years)	27.66 ± 9.65	-	-	28.03 ± 10.80	-	-	27.26 ± 8.34	-	-	0.731
Average length of episode (weeks)	4.07 ± 11.17	0–96.00	2.00	3.13 ± 3.76	0–24.00	3.00	5.09 ± 15.70	0–96.00	2.00	0.523
Average time between episodes (years)	2.80 ± 2.89	0–20.00	2.00	3.02 ± 3.57	0–20.00	2.00	2.59 ± 2.00	0–9.00	2.25	0.529
Numbers of years of education (years)	14.70 ± 2.57			14.56 ± 3.06	-	-	14.84 ± 1.95			0.645
Mood stabiliser dose (mg/day) (Carbamazepine equivalents)	175.05 ± 277.64	0–1000.00	0.00	120.61 ± 231.85	0–800.00	0.00	232.43 ± 311.85	0–1000.00	0.00	0.149
Antipsychotic dose (mg/day) (haloperidol equivalents)	7.50 ± 45.76	0–400.00	0.17	2.16 ± 3.11	0–11.68	0.33	13.13 ± 65.49	0–400.00	0.03	0.970
Number of episodes	3.89 ± 3.17	-	-	4.00 ± 3.48	-	-	3.78 ± 2.83	-	-	0.764
PANSS (Positive subscale)	8.80 ± 2.65	-	-	8.74 ± 2.04	-	-	8.86 ± 3.19	-	-	0.843
PANSS (Negative subscale)	9.61 ± 3.28	-	-	9.74 ± 3.22	-	-	9.46 ± 3.27	-	-	0.708
PANSS (General subscale)	19.36 ± 3.79	-	-	19.56 ± 4.34	-	-	19.14 ± 3.16	-	-	0.625
PANSS (Total score)	37.76 ± 6.52	-	-	38.05 ± 7.16	-	-	37.46 ± 5.85	-	-	0.695
YMRS* total score	1.99 ± 2.42	-	-	1.87 ± 2.47	-	-	2.10 ± 2.38	-	-	0.673
HDRS^@^ total score	3.03 ± 2.13	-	-	2.87 ± 2.12	-	-	3.19 ± 2.15	-	-	0.520
SOFAS*** score	61.76 ± 12.46	-	-	63.13 ± 13.45	-	-	60.28 ± 11.29	-	-	0.326
BMI**	27.03 ± 7.77	-	-	27.31 ± 5.90	-	-	26.74 ± 9.42	-	-	0.753
Monthly income (₦) Naira	46 389 ± 71 060	0–403 000	20 000	33 026 ± 36 189	0–150 000	22 500	61 323 ± 94 658	0–403 000	20 000	0.409
Net worth (₦) Naira	347 486 ± 913 692	0–5 000 000	30 000	310 236 ± 837 175	0–5 000 000	50 000	386 805 ± 998 606	5000–5 000 000	20 000	0.987

SOFAS, Social and Occupational Functioning Assessment Scale; HDRS, Hamilton Rating Scale for Depression; PANSS, Positive and Negative Syndrome Scale; YMRS, Young Mania Rating Scale; BMI, Body Mass Index; Fishers, Fisher Exact Test; PSQI, Pittsburgh Sleep Quality Index; MWW, Mann-Whitney U test; s.d., standard deviation.

**TABLE 1b:** The sociodemographic and clinical characteristics of participants and comparison of clinical features of bipolar disease according to Pittsburgh Sleep Quality Index scores.

Characteristics	*n*	%	PSQI *n* > 5		PSQI ≤ 5		
			*n*	%	*n*	%	
**Gender**							
Male	29	38.2	13	33.3	16	43.2	0.374
Female	47	61.8	26	66.7	21	58.6	-
**Work status**							
Employed	64	84.2	32	82.1	32	86.5	0.596
Unemployed	12	15.8	7	17.9	5	13.5	-
**Marital status**							
Single/Never married	26	34.2	10	26.3	16	43.2	**0.013** (Fishers)
Married/cohabiting	42	55.3	21	55.3	21	56.8	-
Divorced/widowed/separated	7	9.2	7	18.4	0	0.0	-
**Family history of mental illness**							
No	53	69.7	27	69.2	26	70.3	0.921
Yes	23	30.3	12	30.8	11	29.7	-
**Type of antipsychotic**							
Typical antipsychotic	27	35.5	16	64.0	9	36.0	0.425
Atypical antipsychotic	19	25.0	11	52.4	10	47.6	-
**Lifetime suicide thoughts**							
No	53	69.7	28	71.8	25	67.6	0.688
Yes	23	30.3	11	28.2	12	32.4	-
**Lifetime suicidal plan**							
No	64	84.2	36	92.3	28	75.7	**0.047**
Yes	12	15.8	3	7.7	9	24.3	-
**Lifetime suicidal attempt**							
No	71	93.4	37	94.9	34	91.9	0.671 (Fishers)
Yes	5	6.6	2	5.1	3	8.1	-
**Current suicidality**							
Absent	75	98.7	38	97.4	1	2.6	1.000 (Fishers)
Present	1	1.3	37	100.0	0	0.0	-
**Medical comorbidity**							
Present	10	13.2	34	87.2	32	86.5	0.929
Absent	66	86.8	5	12.8	5	13.5	-

Note: Bold indicates *p*-values that are less than 0.05.

Although the differences were not statistically significant, participants with poor sleep quality had a higher average length of an episode, used higher doses of mood stabiliser and antipsychotics per day, had lower social and occupational functioning, lower monthly income and financial net worth than participants with good sleep quality (Tables 1a and 1b).

### Characteristics of sleep quality in bipolar patients

Participants with good quality sleep did significantly better than those with poor quality sleep in the seven components of the PQSI except for habitual sleep efficiency (Component 4) and sleep disturbances (Component 5). Therefore, participants with good sleep quality had higher total sleep duration, lower time to fall asleep (sleep latency), better subjective quality of sleep, were less likely to use sleep medications and had less daytime dysfunction than participants with poor sleep quality (see Tables 2a and 2b).

**TABLE 2a:** Characteristics of sleep quality in bipolar patients.

Characteristics	Mean ± s.d.	Range	Median	PSQI ≤ 5 (*n* = 39)	Range	Median	PSQI > 5 (*n* = 37)	Range	Median	*p*
Total sleep time (Hours)	8.19 ± 1.57	-	-	9.21 ± 1.09	-	-	7.65 ± 1.80	-	-	**0.003**
Time to fall asleep (Sleep latency, minutes)	20.24 ± 16.50	-	-	15.49 ± 10.97	-	-	25.24 ± 19.74	-	-	**0.011**
Habitual sleep efficiency (%)	97.54 ± 7.77	-	-	98.31 ± 3.34	-	-	96.74 ± 10.62	-	-	0.395
**PSQI scores**		-	-		-	-		-	-	
Subjective quality of sleep	0.36 ± 0.60	0–3.00	0.00	0.10 ± 0.31	0–1.00	0.00	0.62 ± 0.72	0–3.00	1.00	**< 0.001**
Sleep disturbances	0.62 ± 0.49	-	-	0.60 ± 0.59	-	-	0.64 ± 0.49	-	-	0.682
Use of sleep medication	1.66 ± 1.46	-	-	0.77 ± 1.33	-	-	2.59 ± 0.90	-	-	**< 0.001**
Daytime dysfunction	0.49 ± 0.79	0–3.00	0.00	0.15 ± 0.37	0–1.00	0.00	0.84 ± 0.96	0–3.00	1.00	**< 0.001**
PSQI global score	4.49 ± 2.85	-	-	2.28 ± 1.38	-	-	6.81 ± 2.04	-	-	**< 0.001**

Note: Bold indicates *p*-values that are less than 0.05.

PSQI, Pittsburgh Sleep Quality Index.

**TABLE 2b:** Characteristics of sleep quality in bipolar patients.

Characteristics	*n*	%	PSQI ≤ 5		PSQI > 5		*p*
			*n*	%	*n*	%	
**Use of sleeping medications in the last month**							
No	32	42.1	29	74.4	3	8.1	**< 0.001**
Yes	44	57.9	10	25.6	34	91.9	-
**Subjective sleep quality**							
Good	73	96	39	100	0	0.0	0.111 (Fishers)
Bad	3	4	34	91.9	3	8.1	-
**Daytime dysfunction**							
No	51	67.1	33	84.6	18	48.6	**0.001**
Yes	25	32.9	6	15.4	19	51.4	-
**Sleep duration**							
Adequate	72	94.7	39	100.0	0	0.00	0.051 (Fishers)
Inadequate	4	5.3	33	89.2	4	10.8	-

Note: Bold indicates *p*-values that are less than 0.05.

PSQI, Pittsburgh Sleep Quality Index.

### Regression analysis

Binary logistic regression analysis to determine the factors that were independently associated with poor sleep quality in euthymic bipolar disorder patients indicated that subjective quality of sleep, use of sleep medication, daytime dysfunction were independently associated with poor sleep quality (see Table 3).

**TABLE 3:** Binary logistic regression analysis to determine factors independently associated with poor sleep quality in euthymic bipolar disorder patients.

Characteristics	OR	*p*	95% CI for OR	
			Lower	Upper
**Marital status**				
Married/cohabiting	1.60	0.18	0.59	4.32
Divorced/widowed/separated	0.19	0.07	0.02	1.77
Single/Never married	1	-	-	-
**Lifetime suicidal plan**				
No	1.80	0.745	0.05	61.66
Yes	1	-	-	-
Time to fall asleep (Sleep latency, minutes)	1.09	0.150	0.97	1.22
Total sleep time (Hours)	0.99	0.094	0.97	1.00
Subjective quality of sleep	60.08	**0.045**	1.10	3289.47
Use of sleep medication	13.36	**0.007**	2.03	88.05
Daytime dysfunction	16.23	**0.043**	1.09	241.63

Note: Bold indicates *p*-values that are less than 0.05.

## Discussion

Nearly half (48.7%) of the euthymic bipolar I patients had poor sleep quality suggesting that many euthymic patients with bipolar disorder display substantial disturbance in sleep quality during remission. This means that about half the population of bipolar disorder patients purportedly in remission are at the risk of neurocognitive impairment, low immunity, obesity, heart disease, diabetes, infertility, poor social and occupational functioning and other effects of poor sleep quality because of impaired sleep quality.^10,11,12,13^ Sleep disturbance is also a trigger for relapses and recurrences. This can lead to a vicious cycle of relapses and poor sleep quality. The result adds to existing reports indicating that euthymic bipolar patients have substantial poor sleep quality even when they are adjudged symptomatically to be in remission.^15,23,24,25,26^ The prevalence of sleep quality in our study (48.7%), is lower than those of existing studies in similar euthymic patients (56.5% – 82.9%).^23,27,28^ Methodological differences could have accounted for the lower prevalence observed in our study.

We found that a significantly higher proportion of the participants who had poor sleep quality were single or never married compared to those with good sleep quality. This is in keeping with existing studies showing that poorer sleep quality is more commonly associated with unpartnered relationship status (single, divorced, cohabiting, widowed) than partnered relationship status (i.e. married or cohabiting).^29,30^ Nevertheless, marital happiness and anxiety are confounders in the associations between sleep quality and unpartnered relationships. Marital happiness is associated with a better quality of sleep in women,^31,32^ while anxiety in close relationships is associated with poorer subjective sleep quality.^33,34^ Such associations suggest that being in a partnered relationship may be beneficial to the health and well-being of patients with bipolar disorder.

We found a significant association between sleep quality and a suicidal plan. This is in keeping with existing studies that found an association between suicidal behaviours and impaired quality of sleep amongst euthymic bipolar patients.^35,36^ Existing studies indicate that there is an association between impaired sleep quality and suicidal ideation, suicidal attempts, and completed suicide.^37^

Furthermore in males, poor sleep quality is associated with suicide attempts, but not in females. This implies that there may be sex differences in the relationship between disturbed sleep quality and suicidal behaviour. Testosterone levels and age have been implicated in this phenomenon.^38,39^ It has been suggested that low testosterone level is associated with suicide in the elderly, while high testosterone level is associated with suicide in adolescents and young adults.^40^

The mean sleep latency in participants with poor sleep quality in our study (25 min) was significantly higher than that for participants with good sleep quality (15 min). Additionally, it was higher than the normal 10–20 min usually found in adults. The findings regarding sleep latency are in parity with existing reports that indicate that euthymic bipolar patients have longer sleep-onset latency than during manic or depressive episodes.^41^

We found evidence of a significant association between poor sleep quality and subjective quality of sleep, use of sleep medication and daytime dysfunction amongst euthymic patients with bipolar disorder. Notably, these associations remained significant after controlling for several covariates in binary logistic regression.

The finding that daytime dysfunction was independently associated with euthymic bipolar patients replicates the report on sleep by Geoffroy et al. conducted amongst euthymic bipolar disorder patients and healthy controls. The authors found poorer sleep efficiency, frequent sleep disturbances and daytime dysfunction to be associated with euthymic bipolar disorder.^42^ Similarly, Cretu et al.^43^ found that bipolar patients in remission compared to healthy controls had significantly worse daytime dysfunction. Earlier studies also showed similar trends, for example, Harvey et al.^25^ showed that daytime dysfunction was poorer in euthymic bipolar patients compared to healthy controls.

Poor sleep quality in euthymic bipolar disorder patients was independently associated with medication use. This is in keeping with existing studies showing that euthymic patients with bipolar disorder are more likely to use sleep medications compared to healthy controls.^43^

There were certain limitations to this study. First is the use of PSQI which is both subjective and retrospective and may be laden with the problem of recall bias. Second, the PSQI generates a global score from its seven component scores. These components are influenced by many factors. Third, the sample size was small; this may have reduced the power of the study to detect significant differences.

## Conclusion

Amongst euthymic bipolar patients, poor sleep quality frequently persists during euthymic periods and can be a focus of targeted intervention. An assessment of sleep quality should be routinely carried out in the assessment of euthymic bipolar patients.
